# Neurofibromatosis type 1 accompanied by acromelanoma: A case report

**DOI:** 10.1097/MD.0000000000041845

**Published:** 2025-03-28

**Authors:** Shuai Dong, Mingrui Zhang, Yuanchen Zhang, Yuan Ma, Yan Mou

**Affiliations:** a Department of Dermatology, The Second Hospital of Jilin University, Changchun, Jilin Province, China; b Department of Dermatology, Women and Children’s Hospital of Ningbo University, Ningbo, China.

**Keywords:** acromelanoma, case report, follow-up observation, interferon injection, neurofibromatosis type 1

## Abstract

**Rationale:**

Neurofibromatosis is an autosomal dominant genetic disorder characterized by developmental abnormalities of the nervous system, skin, and bones. Clinically, it is relatively rare and is often associated with various benign and malignant tumors.

**Patient concerns:**

However, reports of its association with malignant melanoma are scarce, and has been reported as a case report in this study. Case reports and treatment options are discussed below.

**Diagnoses:**

A 71-year-old male presented with long-standing skin nodules and plaques including a growing and itchy black patch on his left heel. Pathological examination revealed neurofibroma in the nodules and malignant melanoma in the heel lesion. The diagnosis was neurofibromatosis type l with acromelanoma.

**Interventions:**

Due to financial constraints, the patient refused surgical excision of the tumor and opted for conservative treatment with interferon injections.

**Outcomes:**

The patient is currently under follow-up observation.

**Lessons:**

This rare case underscores the importance of monitoring genetic disorder patients for tumor risk, emphasizing timely intervention.

## 1. Introduction

Multiple neurofibromatosis, also known as von Recklinghozen’s disease, is an autosomal dominant inherited disease. It is a rare disease that is often accompanied by multiple benign tumors and malignant tumors. However, there are few reports of neurofibromatosis accompanied by malignant melanoma, and no such reports from China. Here, we report a case of a 71-year-old male man who was diagnosed with neurofibromatosis type 1 with acromelanoma. A series of laboratory tests including dermatological examination, histopathological examination of the heel patch and the nodules were performed to confirm the diagnosis. We summarize the clinical features of this patient and the efficacy of the existing treatments, to provide reference for the management and treatment of patients with neurofibromatosis type 1 complicated with acromelanoma. This case is unique in that it is the first known neurofibromatosis type 1 with acromelanoma published in the literature in China.

## 2. Case report

**Figure 1.**
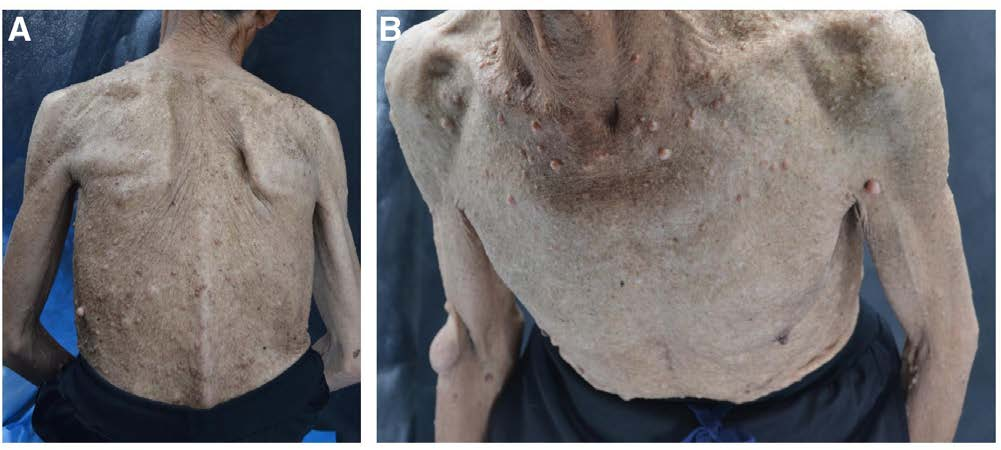
The patient’s body was scattered with soft and sessile or pedicled nodules of varying sizes that were moveable and a light purplish-red color.

The 71-year-old male patient had light purplish-red nodules and brown patches of varying sizes scattered all over his body. These had been present for >60 years and had gradually enlarged and increased in number as he aged. He also had a black patch on his left heel that had been present for >20 years; over the past month, this had significantly increased in size and become pruritic and caused pain when standing. Previous history: The patient had a more than 10-year history of scoliosis and had not been examined to determine its cause and a treatment. Family history: The patient’s sister had approximately 10 nodules that were similar to the patient’s nodules and also had bilateral axillary freckles. However, the patient’s parents and 2 younger brothers did not have similar diseases. Physical examination: The patient was short (height: 162 cm) and thin (weight: 43 kg) and had a lateral curvature in the thoracic vertebrae. The patient was conscious, slightly unresponsive, in an autonomous position, and cooperated with the physician. Dermatological examination: The patient had soft and sessile or pedicled nodules of varying sizes scattered all over his body that exhibited good mobility, were not tender, and were a light purplish-red color (Fig. 1).

**Figure 2.**
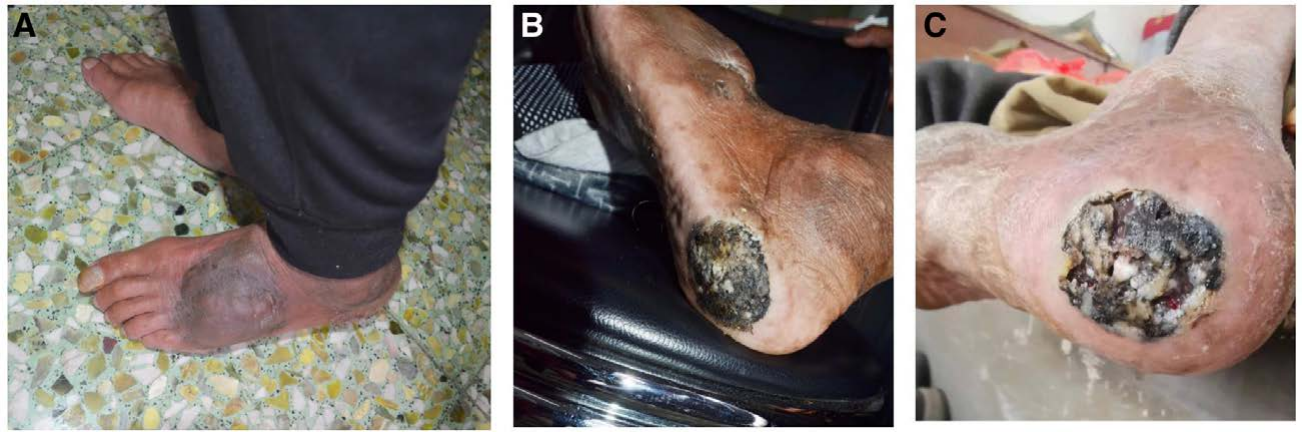
(A) Two dark coffee-colored spots were present on the left dorsal area and heel of the foot. (B) A ~4.0 cm × 3.5 cm black patch with irregular edges and an unclear boundary, and featuring an ulcer, was present on the bottom of the left foot. (C) The area of the left foot was slightly enlarged, and the ulcer was worse than before.

The patient also had 7 spots of various sizes scattered over his body that were irregularly shaped and uniformly pigmented (those on the left dorsal foot and heel of the foot were dark brown [Fig. 2A], whereas the remainder were light brown) and had light-brown freckles in the bilateral axillae. In addition, the patient had an approximately 4.0 cm × 3.5 cm black patch with irregular edges and an unclear boundary on the bottom of the left foot (Fig. 2B).

**Figure 3.**
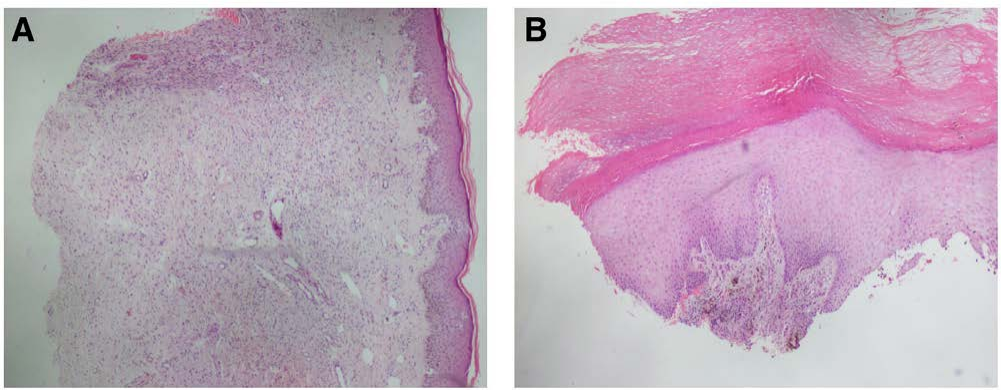
(A) Most spindle tumor cells were disordered and distributed in the lightly stained collagen matrix, the nucleus was deeply stained and S-shaped, and the cytoplasm was weakly eosinophilic. Therefore, the tumor was considered to be a neurofibroma (hematoxylin–eosin [HE] × 100). (B) Heel: The tissue sample was small, and the melanoid tumor cells in the metacarpal and toe skin, stratum corneum, stratum acanthum, and dermis had obvious atypia. Therefore, the tumor was considered to be a malignant melanoma, and in keeping with clinical practice, it was recommended that it be completely resected and then subjected to a pathological examination (HE × 100). HE = hematoxylin–eosin.

Histopathological examination of the nodules: Most spindle tumor cells were disordered and distributed in the lightly stained collagen matrix, the nuclei were deeply stained and S-shaped, and the cytoplasm was eosinophilic. Thus, the tumor was considered to be a neurofibroma (Fig. 3A).

Histopathological examination of the heel patch: The tissue was small, and the melanoid tumor cells in the metacarpal and toe skin, the stratum corneum, the stratum acanthosis, and the dermis exhibited obvious atypia. Thus, this heel patch was considered to be a malignant melanoma, and in keeping with clinical practice, it was recommended that it be completely resected and pathologically examined (Fig. 3B).

Diagnosis: Neurofibromatosis type 1 with acromelanoma.
Treatment: The patient and his family members were fully informed of the condition, and it was recommended that the patient undergo positron emission tomography–computed tomography examination to confirm the presence of metastases and also immediately undergo extensive surgical resection and skin grafting. Due to economic reasons, the patient declined the recommended examination and treatment and instead received interferon injection therapy in a local hospital.
Follow-up: After 6 months of follow-up, the patient was admitted to a local hospital due to abdominal pain. Abdominal computed tomography showed a space-occupying lesion in the colon that was not revealed by colonoscopy. The lesion area of the patient’s left foot was slightly enlarged, and the ulceration was more severe than before (Fig. 2C). No suspicious lesions were observed on other parts of the skin. Symptomatic treatment was given, and the patient was discharged after symptom relief. The patient remains under observation and follow-up.

## 3. Discussion

Neurofibromatosis is a genetic disorder that manifest as abnormal development of the nervous system, skin, bones, and other areas. It is clinically divided into several subtypes, with the 2 most common subtypes being neurofibromatosis type 1 (NF1) and NF2. NF1 is an autosomal dominant neoplastic disease caused by the mutation of the NF1 gene and has an estimated prevalence of 1/4000 to 1/2000.^[1]^ NF1 is located on chromosome 17 (17q11.2) and has a total length of 350 kb. It encodes neurofibromin, which inhibits the rat sarcoma virus/mitogen-activated protein kinase and phosphoinositide-3-kinase/mammalian target of rapamycin signaling pathways and thus reduces cell proliferation and differentiation, thereby inhibiting tumor growth.^[2]^ Mutation of NF1 leads to a deficiency of neurofibromin, resulting in an excess of the active form of rat sarcoma virus–guanine triphosphatase, which promotes excessive growth of cells, resulting in nerve and skin damage^[3]^ and various benign and malignant tumors. NF1 is inherited in an autosomal dominant manner with 100% penetrance,^[4]^ and there is a 50% prevalence of NF1 in the offspring of those whose NF1 is caused by germ cell variation. Compared with the risk of NF1 in a proband who has an established pathogenic mutation, the risk of NF1 in a proband sibling who has a new mutation is higher due to the possible occurrence of gonadal mosaicism.^[5]^ In this case, the patient’s parents and grandparents did not have similar symptoms to the patient but his sister did, and it was speculated that this was attributable to gonadal mosaicism. Patients may exhibit young- or late-onset NF1, and the disease has diverse clinical manifestations. These can include coffee-milk plaques (typically denoted “café-au-lait macules”) and multiple neurofibromas, which often co-occur with multiple system involvement, manifesting as a variety of benign and malignant tumors; abnormalities in the bones, endocrine system, and nervous system; and cardiovascular and cerebrovascular diseases. In 2021, the 7 diagnostic criteria of NF1 were updated,^[6]^ and if 2 or more of these criteria are met, a diagnosis of NF1 can be made. In this case, the patient’s appearance, clinical manifestations, and skin pathological results allowed a definitive diagnosis of NF1 to be made.

Malignant melanoma is a rare malignant tumor in China, but it has a high fatality rate and its incidence is increasing. There are 4 important subtypes of malignant melanoma, namely malignant lentigoid melanoma, superficial spreading malignant melanoma, acromelanoma, and nodular malignant melanoma.^[7]^ Acromelanoma is the most common subtype in Asian people^[8]^; it usually occurs in those aged 50 to 60, and has an equal incidence in men and women. Acromelanomas typically grow on the fingers or toes in weight-bearing areas and exhibit a short in situ growth period followed by aggressive bidirectional (horizontal and vertical) growth.^[9]^ Lesions initially appear as patches with irregular and blurred edges and gradually develop into blue or black nodules, which may be ulcerated. In this case, a definitive diagnosis was made by skin histopathology, and gross observations were consistent with the typical observable manifestations of acromelanomas. In addition, it is highly likely that the abdominal symptoms that the patient presented with at follow-up are indicative of tumor metastasis.

Neurofibrin is a negative regulator of neural crista-derived tissue growth and differentiation, and thus, patients with NF1 are at increased risk of developing neural crista-derived tumors.^[10]^ However, although melanocytes originate from the neural crest and NF1 is frequently mutated in sporadic melanomas,^[11]^ and previous case reports have suggested the presence of a relationship between melanoma and NF1, this has not been confirmed by large population-based studies. For example, a large study by Uusitalo et al^[12]^ in Finland found no statistical evidence of an increased prevalence of melanoma in those with NF1, that is, only 3 of 14,404 patients with NF1 had melanoma (prevalence = 0.21%), and other, smaller studies have suggested that the prevalence of melanoma is 0% to 5.5% in those with NF1.^[13]^ The lack of strong evidence for an increased risk of melanoma in those with NF1 may be due to the decrease in sun exposure in this population. That is, although patients with NF1 are generally not advised to avoid sunlight, they may do so because of physical and/or mental disabilities, and social isolation. Johnson et al^[14]^ found that compared with children without NF1, those with NF1 participate less in various social activities, that older people with NF1 often feel lonely, and that many people with NF1 may have a life situation that leads to reduced sun exposure, all of which may offset the increased risk of melanoma caused by NF1 mutations. However, further research is needed to test this hypothesis.^[13]^ Nevertheless, approximately 90% of Caucasian people with NF1 have cutaneous primary melanomas, usually on the back, chest, abdomen, and lower extremities, and sun exposure plays an important role in their development.^[15]^ In contrast, in Asians and other people of color with NF1, cutaneous primary melanomas usually appear on the plantar fascia, toes, ends of fingers, subnails, and other extremities, and their appearance is weakly correlated with sun exposure. Accordingly, the author believes that a large-scale study of the latter population would reveal the relationship between melanoma and NF1; however, such a study has yet to be performed. In addition, people with NF1 may have difficulty distinguishing other skin masses from café-au-lait macules and neurofibromas and may ignore the occurrence and development of other skin diseases. For example, the patient described herein has a long history of foot plaques and did not present until he was experiencing pruritus and pain, resulting in a delay in treatment.

## Author contributions

**Conceptualization:** Shuai Dong.

**Data curation:** Mingrui Zhang.

**Formal analysis:** Mingrui Zhang.

**Methodology:** Yuanchen Zhang.

**Writing – original draft:** Yuan Ma.

**Writing – review & editing:** Yan Mou.
